# Metabolic Maturation of White Matter Is Altered in Preterm Infants

**DOI:** 10.1371/journal.pone.0085829

**Published:** 2014-01-22

**Authors:** Stefan Blüml, Jessica L. Wisnowski, Marvin D. Nelson, Lisa Paquette, Ashok Panigrahy

**Affiliations:** 1 Department of Radiology, Children’s Hospital Los Angeles, Los Angeles, California, United States of America; 2 Division of Neonatology, Children’s Hospital Los Angeles, Los Angeles, California, United States of America; 3 Dornsife Cognitive Neuroscience Imaging Center, USC, Los Angeles, California, United States of America; 4 Rudi Schulte Research Institute, Santa Barbara, California, United States of America; Robert Debre Hospital, France

## Abstract

Significant physiological switches occur at birth such as the transition from fetal parallel blood flow to a two-circuit serial system with increased arterial oxygenation of blood delivered to all organs including the brain. In addition, the extra-uterine environment exposes premature infants to a host of stimuli. These events could conceivably alter the trajectory of brain development in premature infants. We used *in vivo* magnetic resonance spectroscopy to measure absolute brain metabolite concentrations in term and premature-born infants without evidence of brain injury at equivalent post-conceptional age. Prematurity altered the developmental time courses of N-acetyl-aspartate, a marker for axonal and neuronal development, creatine, an energy metabolite, and choline, a membrane metabolite, in parietal white matter. Specifically, at term-equivalency, metabolic maturation in preterm infants preceded development in term infants, but then progressed at a slower pace and trajectories merged at ≈340–370 post-conceptional days. In parieto/occipital grey matter similar trends were noticed but statistical significance was not reached. The timing of white matter development and synchronization of white matter and grey matter maturation in premature-born infants is disturbed. This may contribute to the greater risk of long-term neurological problems of premature infants and to their higher risk for white matter injury.

## Introduction

The most significant physiological switch at birth is the transition from fetal parallel high systemic/low pulmonary blood flow to a two-circuit serial system and the increased arterial oxygen content of blood delivered to all organs including the brain. Additionally, in the extra-uterine environment most premature neonates experience respiratory distress and are exposed to a host of physiological stimuli ranging from medications to infectious pathogens to increased sensory-motor stimulation. Moreover, the lack of fetal-placental circulation also means that key factors from placental or maternal circulation, including hormones, may not be delivered to the developing brain. These events occur irrespective of the developmental stage of the neonate and individually or collectively could conceivably alter the trajectory of brain development in premature-born neonates and contribute to the increased risk for long-term neurodevelopmental disorders in this population [1], [2].

Accompanying the morphological and functional changes is the biochemical maturation of the brain. Using non-invasive *in vivo* magnetic resonance (MR) spectroscopy several groups have reported rapidly changing concentrations of metabolites particularly in the first few months of life [3]–[5]. Changing concentrations of these metabolites may relate with well-described developmental processes. For example, the chemical N-acetyl-aspartate (NAA) is synthesized by and mainly stored in *mature* neurons and axons [6]. Its concentration is thus proportional to the number and/or density of “adult-type” neurons and axons and its rapid increase in early brain development is a surrogate measure of axonal growth and neuronal maturation. Other metabolites that can be measured include creatine (Cr), an energy metabolite used by brain cells to replenish ATP, choline (Cho), a membrane metabolite, and glutamate (Glu), the most abundant excitatory neurotransmitter of the brain [7].

We hypothesized that in the absence of overt injury, the physiological and stimulatory changes following premature birth alter the underlying tissue biochemistry–as such, brain development–, potentially in a regionally or tissue-specific manner. To test this, we compared metabolism in white matter (WM) and grey matter (GM) regions in preterm and term infants in a cross-sectional study without evidence of brain injury at equivalent post-conceptional (PC) ages. Finally, to assess whether there was a “dose-dependent” effect of prematurity on the biochemical maturation of WM or GM, we compared metabolism in these same regions in the most extreme preterm infants (born at <28 weeks PC age) to the later preterm-born and term-born infants.

## Materials and Methods

### Ethics Statement

The Children’s Hospital Los Angeles Committee on Clinical Investigations approved the review of already existing data that were acquired as part of the clinical work-up and the requirement for signed consent by the parents was waived.

### Subject Selection

MR examinations and medical records of 656 patients aged between 270 (term) –370 PC days (∼ 3.5 months) referred for brain MR studies at Children’s Hospital Los Angeles between 2001–2011 were reviewed. Indications that warranted clinical MR studies of these infants included prematurity itself due to the higher risk for WM injury (but not confirmed) and/or sepsis, necrotizing enterocolitis, seizures, meconium aspiration, subdural bleeds, rule-out of infections or inborn errors, to assess brain morphology, poor feeding, retinoblastoma, and others. From this group, only infants with no reported abnormalities on MR imaging (i.e., no diffusion abnormalities, no cerebral malformations, large intracranial hemorrhages, infarcts) were selected. For consistency, all potentially eligible MR imaging studies were then centrally re-reviewed by two authors (AP, JLW) and only studies (preterm- and term-born) *without any evidence* of intracranial abnormalities including also extra-axial fluid accumulation, ventriculomegaly, punctate WM lesions, and abnormal T2-signal (i.e., diffuse excessive high signal intensity [DEHSI]) were retained. Subsequently, MR spectroscopy data of poor quality were removed using objective, previously defined, criteria [3]. Furthermore, to exclude infants with significant neurological disorders or developmental delay, a neonatologist (LP), a neuropsychologist (JLW), and a pediatric neuroradiologist (AP) reviewed six months of clinical records for each subject. Growth charts for the premature cohort are shown in **Fig. S1**. Finally, although the cutoff of 37 weeks is well-regarded as an indication of term birth, to generate a better separation of our preterm- and term-born infants, we excluded infants born between 37 and 39 weeks (<39 weeks, 0 days) from our analyses. These procedures yielded a final sample of 81 infants: 51 term-born and 30 premature-born, which entered the final analysis (**Table 1**).

**Table 1:** A total of 171 spectra obtained from three different brain regions from 81 infants are included in the analyses.

		Term	Preterm	All
Infants		51	30	81
GA^a^		40.0±0.5(39–41)	29.8±4.6(23–36)	
PCA^b^		44.6±3.4(40.1–52.7)	45.7±4.1(38.6–53.6)	
	pWM	45	27	73
Number ofStudies	GM	41	22	63
	fWM	24	12	36
		110	61	171

^a^ Gestational age (in weeks) at birth (mean±SD (range)),

^b^ post-conceptional age (in weeks) at the time of the MR examination (mean±SD (range)).

### Brain Regions

**Figure 1:**
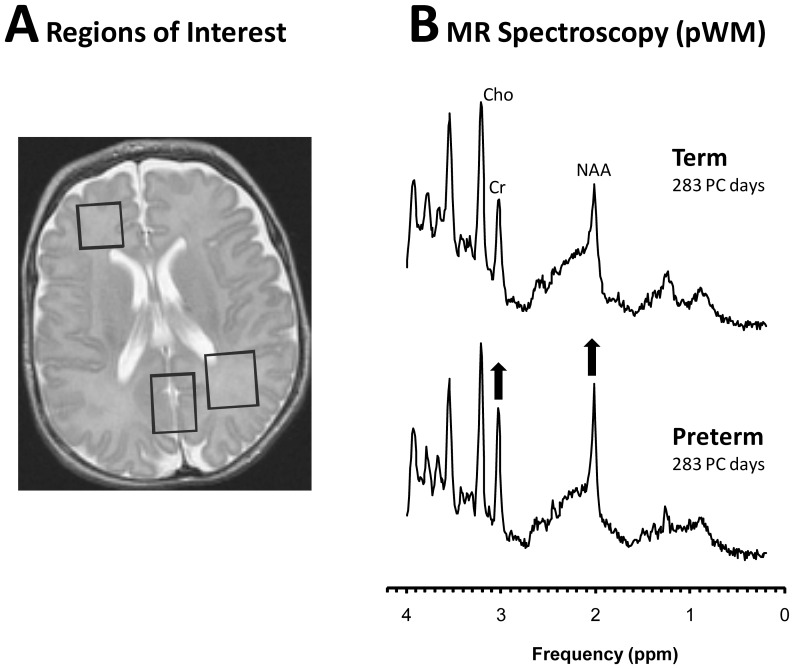
MR images and ^1^H MR spectra of term-born and premature-born infants. T2-weighted MR imaging of the newborn brain indicating the regions of interest in parietal WM, parieto/occipital GM, and frontal WM from where spectra were acquired for this study (**A**). In representative parietal WM spectra of term-born and premature-born infants at equivalent post-conceptional (PC), NAA and Cr are visibly more prominent in the preterm spectrum (**B**). When data from all infants are analyzed, Cho is also significantly different between the two groups.

Biochemical profiles of three distinct brain regions (**Fig. 1**) were analyzed in detail. 1) The first region of interest (ROI) contained mostly *parietal white matter*, dorsolateral to the trigone of the lateral ventricle. Enclosed in this ROI are the longitudinal association tracks including the superior longitudinal fasciculus (anterior-posterior); cingulum (anterior-posterior; medially); and the centrum semiovale; also short U-fibers and white matter extending into parietal gyri). 2) The second ROI contained mostly parietal/occipital *grey matter*. It included the precuneus, posterior cingulate and extending inferiorly into retrosplenial cingulate and posteriorly into cuneus (medial occipital lobe; supracalcarine). 3) In a smaller number of infants spectra were also acquired of mostly *white matter* of the *prefrontal lobes*, dorsolateral to the anterior horn of the lateral ventricle, which included longitudinal association tracts, frontothalamic tracts, short U-fibers (connecting adjacent gyri), and white matter in the frontal gyri.

### Data Acquisition and Processing

All MRS studies were performed on a 1.5T MR system (Signa LX, GE Healthcare, Milwaukee, WI), and were acquired as part of clinically indicated MR examinations. All spectra were acquired using a single-voxel point resolved spectroscopy (PRESS) sequence with a short echo time of 35 milliseconds, a repetition time of 1.5 seconds, 128 signal averages, and a total acquisition time for each spectrum of approximately five minutes, including scanner adjustments. The sizes of the ROIs were typically 3 cm^3^ and a custom-designed newborn head coil was used to ensure an optimal signal-to-noise ratio. Processing was performed with commercially available LCModel software (Stephen Provencher Inc., Oakville, Ontario, Canada, LCModel Version 6.1-4F) and was fully automated. Concentrations of NAA, Cr, Cho, myo-Inositol (mIns), Glu, and taurine (Tau) were determined. Metabolite concentrations were corrected for varying fractions of cerebrospinal fluid in the selected ROIs [8]. For absolute quantitation, the unsuppressed water signal was used as a concentration reference [37]–[43] as illustrated in **Fig. S2**.

### Modeling of Metabolite Concentration versus PC Age in Term-born Infants

Linear regression lines as well as sigmoid functions were used to parameterize the time courses. The functions and parameters that provided the best fits (minimum χ^2^) for each metabolite and brain region are provided in **Table S1**. Least-squares fits were carried out using MATLAB (R2010b, The Mathworks, Inc., Natick, MA, USA). Errors for the fitted parameters were determined by Monte Carlo simulation [9].

### Statistical Analyses

**Figure 2:**
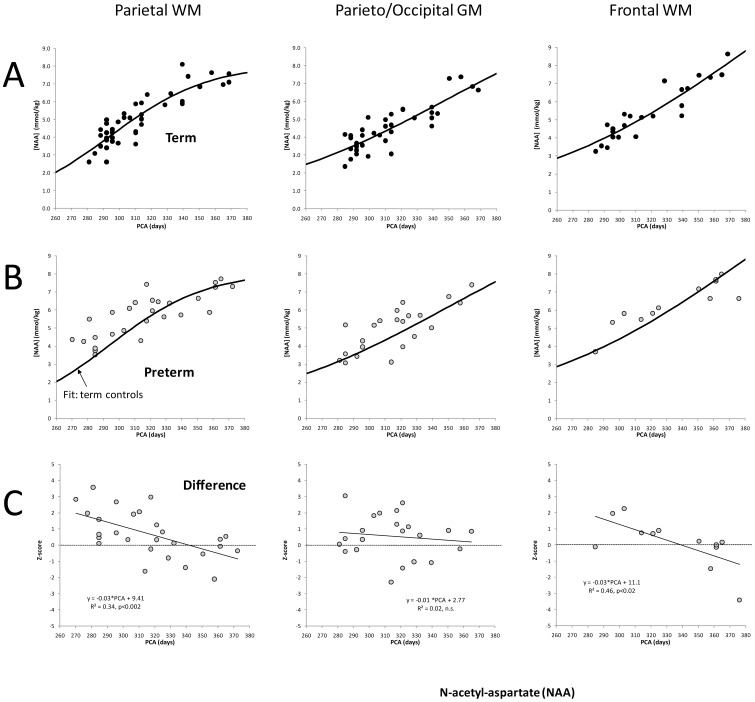
NAA concentrations versus post-conceptional age. Shown are NAA concentrations versus PC age of *term* newborns in parietal WM, parieto/occipital GM, and frontal WM with the curves that provided the best fits superimposed to the data (**A**). Note the different shape of the curves. Whereas the time of the fastest increase of NAA in parietal WM is around 295 PC days, the fasted increase of NAA in GM and frontal WM is observed 2–3 months later at around 350 and 390 PC days. NAA is believed to indicate axonal growth and neuronal maturations and these data are consistent with developmental processes in parietal WM preceding changes in GM or frontal WM. When NAA concentrations of premature infants are plotted (**B**), a discrepancy is readily noticed for parietal WM (note that the fitted curve for term infants was carried over from figure **A** and that there are more points above than below that curve) but is not observed for parieto/occipital GM and is also less apparent for frontal WM where fewer data points are available. When the normalized difference (z-score) is analyzed, the majority of the preterm data in parietal WM are positive, i.e. at equivalent PC age concentrations are higher in premature brain. However, the differences in parietal WM decrease with increasing PC age. Indeed, at around 340 PC days there is no difference between term and premature brain in respect to NAA concentrations in parietal WM. It appears that frontal WM follows parietal WM. Albeit the mean difference between term and preterm infants is not significant, there is a significant negative slope comparable with the observation made for parietal WM (**Tab. 2**). PC = post-conceptional.

Three statistical analyses were performed. First, the normalized differences (z-scores) between the fitted curves of term infants and measurements in term and in preterm infants were analyzed (**Fig. 2**). For *term* infants this calculation yielded by definition average z-scores = 0 with a standard deviation of ±1 for all metabolites. To determine then whether the preterm infants differed from the term infants, on average, a single-sample two-tailed t-test was used to test whether the mean z-scores in the premature infants were different from zero. Secondly, z-scores were plotted versus PC age and a linear regression analysis (StatPlus, AnalystSoft Inc., Alexandria, VA) was performed to determine whether there was a significant slope. E.g. a slope significantly different from zero would indicate a different pace of biochemical maturation in premature brain versus term brain. Finally, we subdivided the premature group in two subgroups with the first group only including infants born at gestational age (GA) <28 weeks (extreme preterm) and the second group comprising infants with 28≤ GA <37. Analysis of covariance was used to determine whether metabolite changes were different in the two subgroups of premature-born infants. This analysis was performed for parietal WM and parieto-occipital GM. The number of frontal WM spectra from premature infants was not considered sufficient for this detailed analysis.

## Results

### Metabolic Changes in *Term* Infants

**Figure 3:**
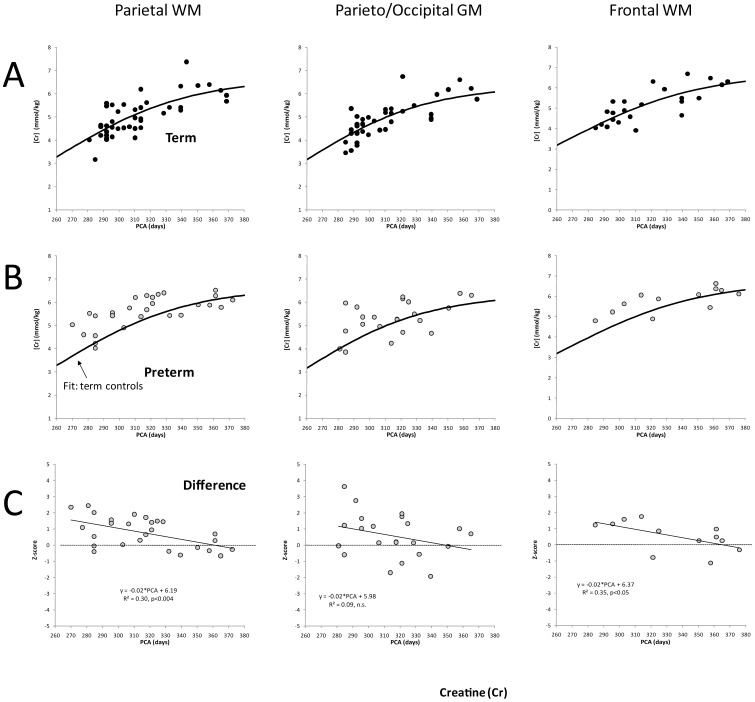
Creatine concentrations versus post-conceptional age. Unlike for NAA, where parietal WM shows a significant different time course when compared with GM and frontal WM, the times courses for the three brain regions are very comparable (**A**). Similar with the observations for NAA, Cr concentrations in premature infants are generally higher than in term-born infants, particularly in parietal WM (**B**). As for NAA, the difference between term and preterm infants decreases with age (**C**).

Concentrations of NAA, Cr, and Glu increased, mIns decreased, and Cho and Tau remained close to constant within the first three months of life in term-born infants as reported earlier by several groups [3]–[5]. However, with more infants included than in previous studies more detailed developmental trends for certain metabolites emerged. For example, the increase of NAA in parietal WM preceded significantly the increases in parieto/occipital GM and frontal WM (**Fig. 2A** **, Table S1**). On the other hand, the time courses of Cr were comparable in the three regions studied (**Fig. 3A**). Similarly, Cho, mIns, and Tau time courses were comparable in the three regions examined. Glutamate, on the other hand, showed the same trend as NAA with an initially faster increase in parietal WM than in frontal WM. The increase of Glu in GM also appeared to lag behind the increase observed in parietal WM.

### Term vs. Preterm Metabolism at Equivalent PC Age

*Average concentrations:* In parietal WM, mean z-scores for NAA (p<0.05), Cr (p<0.001), and Cho (p<0.01) were all significantly different from zero indicating that on average the concentrations of these metabolites were altered in the preterm infants compared to the term infants. No significance differences were detected for mIns, Glu and Tau (**Table 2** **,** **Fig. 2** **–** **4**). In contrast to parietal WM, in parieto/occipital GM, albeit similar trends were observed, none of these six metabolites showed a significant difference between term and premature infants. In frontal WM, where fewer spectra were available for evaluation, only Cho (p<0.05) was significantly different between preterm- and term-born infants. However, trends were consistent with parietal WM with NAA and Cr approaching significance.

**Figure 4:**
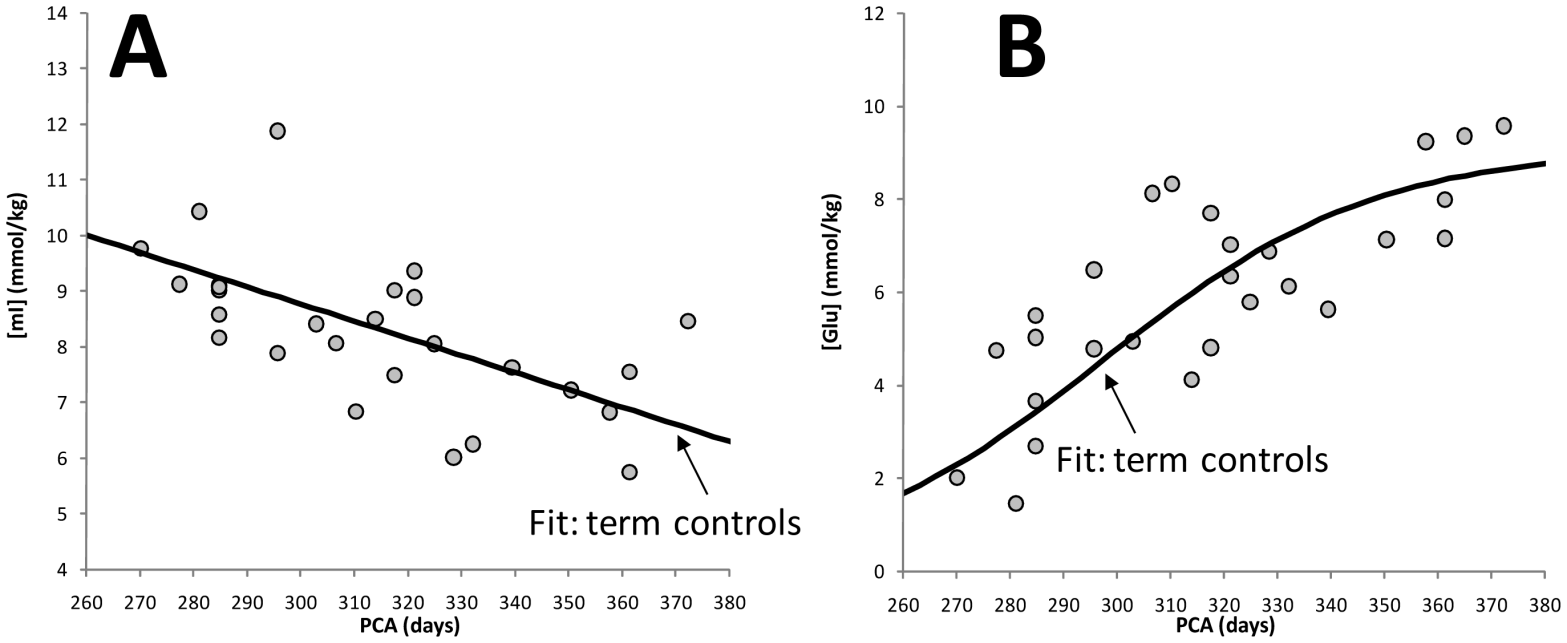
Concentrations of myo-inositol and glutamate versus post-conceptional age. This figure shows the best fit (solid line) of the term data for mIns and glutamate (Glu) with the individual measurements in preterm infants superimposed in parietal WM. There are no apparent differences between term and preterm infants at equivalent PCA. These graphs should be compared with Figs. 2B and 3B (left columns) that illustrate the differences observed for NAA and Cr in parietal WM.

**Table 2:** Summary of z-scores and z-score slopes of preterm infants.

		z-score	z-score slope
	NAA	0.67±1.43*	−0.028±0.002**
Parietal WM	Cr	0.76±0.95***	−0.017±0.001**
	Cho	0.90±1.46**	−0.016±0.003
	NAA	0.55±1.34	−0.007±0.003
Parieto/occipital GM	Cr	0.58±1.36	−0.017±0.003
	Cho	0.47±1.66	−0.009±0.003
	NAA	0.15±1.49	−0.033±0.002*
Frontal WM	Cr	0.53±0.92	−0.017±0.003*
	Cho	1.17±1.66*	−0.021±0.005

The z-scores measure whether the average metabolite levels in term versus preterm infants were significantly different. The z-score *slopes* measure whether an increase (or decrease) of a metabolite concentration in the premature cohort over time was significantly different from what was observed in the term-born infants. E.g., the significantly negative z-score *slope* for NAA in parietal WM indicates that NAA increased slower with age in the premature group. Values for mIns, glutamate, and taurine were all not significantly different from zero and are not listed.

p<0.05, **p<0.01, ***p<0.001.

*Time courses of metabolites:* In addition to the overall differences described above, there were also differences in the *rate* of increase for NAA and Cr in parietal WM. Specifically, while concentrations of NAA and Cr for the preterm infants were initially elevated relative to term born infants at term-equivalency, the rate at which NAA and Cr concentrations increased with age was significantly smaller (both p<0.01) in the preterm-born infants compared to the term-born infants such that at approximately 350 PC days concentrations of NAA and Cr term and preterm-born infants were comparable (**Figs. 2C** and **3C**). In contrast, no significantly different time courses were observed in parieto/occipital GM. For frontal WM, consistent with parietal WM, NAA and Cr also increased significantly slower (both p<0.05) in preterm infants than in term-born infants (**Table 2**).

### Extreme Prematurity (GA <28 Weeks)

Average NAA levels in parietal WM of extreme premature-born infants (GA at birth <28 weeks, n = 10) were not different from levels observed in later preterm-born infants (28≤ GA <37, n = 17). However, the rate of increase was significantly different (p<0.05) with a faster increase observed in later preterm infants closer to the time course seen in term infants. In parietal WM, Cr showed a similar trend with a faster increase in later preterm infants, albeit, the difference in the slopes did not reach significant levels. No significant differences were noted for other metabolites.

## Discussion

Many prematurely born children will lead a normal life; however, they are, nevertheless, at an increased risk for neurodevelopmental problems when compared to children born full-term. These risks increase with decreasing gestational age (and weight) at birth and are particularly high for children born before 28 gestational weeks [2]. Previous imaging studies of premature infants have demonstrated that various patterns of injury (e.g., intraventricular hemorrhage and WM injury) detectable on anatomical MR imaging or cranial ultrasound are factors for long-term neurocognitive outcomes [10]–[12]. However, these patterns of injury do not entirely account for adverse neurocognitive problems in this population. Furthermore, subgroups of premature-born children who go on to suffer from neurodevelopmental disorders have normal appearing anatomical MR scans near term-equivalency [11], [13], [14]. Albeit some of these children may develop problems only at school age, the period between premature birth and the first few months of life may be particularly critical because many developmental processes such as axonal and dendritic outgrowth, neuronal differentiation, synapse formation, and myelination are initiated in a highly synchronized fashion during early brain development [15]–[17].

The main finding of this study was that the synchronization of *metabolic maturation* in the WM and GM was disturbed in premature-born infants compared to term-born infants drawn from the same neonatal population. Physiological changes and/or stimulatory events may have triggered an indeed premature or “false” start of some maturational processes predominantly in WM followed then by a slower than normal progression. In contrast, GM metabolism was not or was less affected by prematurity. In addition, within WM, some metabolites were clearly impacted by prematurity, whereas others were not. This may indicate that the timing and synchronization of developmental processes *within* WM is also affected by prematurity. Furthermore, our data are consistent with a more substantial metabolic desynchronization in more extreme premature infants (GA at birth <28 weeks).

When compared with recent MR imaging studies our finding of an early start of WM *metabolic* maturation in premature infants was unexpected. Most imaging studies of brain maturation in preterm infants near term-equivalency have, if anything, suggested that preterm brain development lags behind term brain development at equivalent PC ages [18]–[21]. These patterns of developmental lag have been most robustly demonstrated in the most extreme preterm cohorts and in preterm infants with severe injury visualized on conventional imaging [11], [12]. Yet, even in studies where preterm infants with documented injury have been excluded, it has been suggested that WM maturation still lags, documented as decreased WM volume and abnormal microstructure [22], [23] albeit there has been variability as a more recent study reported an early onset of WM maturation in preterm infants [24], [25]. Studies of GM maturation in preterm infants have revealed a decrease in GM volume [19] and differences in tissue microstructure [26], [27]. However, most of these studies have included not only high-risk infants, but also infants with WM injury as documented by conventional MRI. It is thus conceivable that, absent overt WM injury, prematurity has less of an impact on GM, which would also be consistent with post-mortem studies [28] and animal models [29].

### Metabolism and WM Maturation in Term and Preterm Infants

Both, NAA and Cr were similarly affected by prematurity in parietal WM as they were both generally higher in the premature cohort at term-equivalent age but then increased at a slower rate than in term-born infants with the concentrations reaching equal levels at approximately 350 PC days (≈2.3 months-old term infant). However, it is likely that the structural or physiological events that relate with cerebral NAA and Cr concentrations are different since their role, synthesis/degradation, regulation, and compartmentalization in the brain are quite different. NAA is mainly stored in *mature* neurons and axons, and is thus generally believed to be a surrogate indicator for axonal outgrowth in WM and neuronal maturation in GM [6]. Cr, on the other hand, is not restricted to the neuronal/axonal compartment but is present in all cells that utilize creatine kinase including astroglia. NAA is synthesized in the mitochondria of neurons, diffuses along the axoplasma, and is degraded in oligodendrocytes, where it does not reach high concentrations, by aspartoacylase providing acetyl groups for myelin lipid synthesis [6]. Cr is synthesized outside the brain in the kidney and liver and transported to the brain where it functions as an energy metabolite by buffering the supply of cellular ATP [30].

In addition, we observed in the *term* cohort, that the NAA increase in parietal WM preceded the increase in frontal WM and parieto/occipital GM. This is consistent with an earlier axonal outgrowth and WM development in parietal brain when compared with frontal brain or with neuronal development in the parieto/occipital brain [15]. In contrast, the Cr time courses in *term* infants in the three regions examined were indistinguishable, with no apparent correlation with the order of development.

With the above in mind, elevated Cr may reflect generally higher energy metabolism in premature brain at equivalent PC age. With the physiological switches occurring at birth, more oxygen becomes available enabling increased mitochondrial oxidation of acetyl-CoA in the tricarboxylic acid (TCA) cycle and the production of more ATP. For the observed higher NAA in the preterm population at equivalent age there are at least two possible explanations, individually or in combination. First, higher NAA may indicate higher axonal density in premature brain due to early axonal outgrowth triggered by altered physiological conditions and stimulatory challenges. A reduction of non-NAA containing cell types would also result in a net increase of the measured NAA concentration over a volume. However, we consider this less likely as we would expect some type of structural abnormalities such as a general loss of tissue and excessive CSF in our preterm cohort, which was not observed. Second, NAA is synthesized in neuronal mitochondria and possibly coupled with mitochondrial activity [6]. With increased mitochondrial activity due to stimulatory challenges and physiological switches at premature birth, more NAA may be synthesized. On the other hand, NAA degradation by aspartoacylase in oligodendrocytes may not be equally increased in the unmyelinated brain [6] creating a bottleneck and NAA may accumulate in the axoplasm at higher concentrations. It should be noted that, clinically, NAA is often used as surrogate measure of axonal/neuronal loss/damage. Specifically, diseases or conditions that are associated with axonal or neuronal loss exhibit reduced levels of NAA. Intuitively, it would be expected that prematurely born infants who are at high risk for injury such as periventricular leukomalacia would show reduced NAA. Indeed, this was observed in preterm infants with punctate WM lesions [31]. In contrast, in this study of preterm infants with normal appearing WM, an increase of NAA was observed instead.

Choline, which was also altered in the WM of premature brain, is a complex peak comprising indistinguishable signal from mostly phosphocholine and glycerophosphocholine. These metabolites are involved in the synthesis and breakdown of membrane phospholipids. Relating Cho to developmental processes is challenging since phosphocholine is initially high in the newborn brain but then decreases whereas glycerophosphocholine is initially low but then increases in the developing brain [32].

### Prematurity and WM Vulnerability

For more than a century, it has been known that the WM of premature infants, particularly in the parietal lobe, consistent with our parietal WM ROI, is especially vulnerable to injury [33], [34]. A range of mechanisms have been proposed including hypoxic-ischemic injury due to immature vasculature and resulting in the release of excitotoxins, infection/inflammation, and free radicals. *None* of the infants included in this study (term or preterm) had any evidence for WM abnormalities or any other abnormalities on MR images. Still, the alterations in metabolism observed in our premature cohort are potentially relevant for increased WM vulnerability. An early or false start of maturational processes and increased energy demand could increase the sensitivity of the WM to ischemia, and accelerate production of reactive oxygen species and free radicals before the development of defense mechanisms (Mn-superoxide dismutase; Cu-Zn superoxide dismutase) [35], which in turn could damage developing WM. Thus, generally, our observations support the idea of a developmental mismatch and the unpreparedness of particularly WM “for the transition from a hypoxic intrauterine to an oxygen-rich postnatal environment” [35].

### Previous MR Spectroscopy Studies of Prematurity

Our findings are not consistent with two earlier studiers that did not report a significant impact of prematurity on brain development [21], [36]. However, the number of infants in the study by Kreis *et al.* was small (5 premature infants and 11 term infants at 41 PC weeks) and did not include extreme prematurity, which rendered their study less likely to detect the impact of prematurity. In the study by Hüppi PS *et al.,* Cr was used as an internal concentration reference. I.e., it was assumed that Cr is constant in term and premature infants. Our study showed that this assumption may not be valid as Cr as significantly different in the two cohorts. However, our observations are generally consistent with a study where metabolism of near term *in utero* fetuses was compared with premature neonates imaged at equivalent PC age [25]. Higher levels of NAA and Cr were observed in premature neonates compared with fetuses. On the other hand, they also reported higher levels of mIns in premature infants, which we did not observe in our cohorts.

### Potential Limitations of the Study

Regions of interest were selected to maximize the amount of WM and GM to the extent technically feasible, however, should not be considered “pure” WM or GM regions but instead as “mostly” WM or “mostly” GM regions. This poses some limitations for the interpretation of the results and adds ambiguity. Not yet significant trends in GM (similar to significant trends in WM) could be explained by the contribution from small amounts of WM that is unavoidably enclosed in our GM location. Alternatively, GM may also be affected by prematurity but to a smaller extent than WM. In any case, it is nevertheless demonstrated by our study that there are significant differences between the impact of prematurity on WM and GM development.

The preterm- and term-born infants studied here were derived from the same population at a single tertiary care children’s hospital. It could be argued that it would have been better to recruit the term-born infants from the community rather than from the same inpatient hospital population. However, by deriving our preterm-born and term-born infants from the same hospital-based neonatal population, it allowed us to more closely match our preterm and term infants to each other. As expected, our preterm-born infants had markedly higher incidences of ventilatory support. Still, with the same stringent subject selection criteria applied to more than 650 patient studies acquired over a 10-year span, two cohorts of infants without any visible patterns of brain injury documented on MR imaging or neurodevelopmental disorder on short-term (i.e., 6 month) follow-up were obtained. This approach then afforded the opportunity to consider the critical impact of prematurity apart from overt brain injury on brain metabolism.

### Conclusions

The biochemical maturation of WM is significantly different in preterm infants compared to term infants whereas no significant differences were observed for GM. The untimely biochemical maturation in WM may contribute to why WM appears more susceptible to injury in preterm infants compared to term infants or even fetuses at equivalent age. At the same time, the timing and synchronization of WM and GM maturation is disturbed. This may contribute to aberrant brain development that underlies the adverse neurocognitive outcomes that develop in this population. Therapeutic interventions that aim to alleviate the possible adverse impact of prematurity on brain function may need to consider the impact of prematurity on both the nature and synchronization of critical maturational processes.

## Supporting Information

Figure S1 **Growth curves for prematurely born subjects.** To rule out the possibility that the accelerated WM maturation in the preterm infants was merely a reflection of accelerated somatic growth, we examined serial measurements of head circumference, weight, and body length, recorded from the preterm infants’ medical records. On average, these three measures remained below the expected normal values, but in line with the expected rate of growth (i.e., slope) throughout the study period (i.e., 60 weeks). There was no evidence for a systematic clinical problem with e.g. the head circumference falling further behind over time. This was expected as subjects were selected based on unremarkable clinical follow-up. Note that the number of individual data points exceeds the number of subjects since several measurements (typically weekly) of head circumference (left), weight (center), and body length (right) were taken. The thick solid lines indicate the 50-percintile lines for normal growth (©2003 Fenton TR; licensee BioMed Central Ltd., http://www.biomedcentral.com/1471-2431/3/13). The thin lines represent the best fit of measurements in the preterm cohort.
(TIF)

Figure S2 **Water concentration.** When studying newborns it needs to be considered that brain water concentrations change with age from ≈ 88% at birth to ≈ 85% at six months of age [37]–[40]. However, it is conceivable that brains of term and preterm infants do have *different* water contents at *equivalent* PC age. To *a priori* exclude that differences in water content could possibly be a factor for systematically different metabolite concentrations, we used an approach that does *not* utilize the age of a subject as the determining factor for the water concentration. Instead, we used the tissue transverse (T_2_) relaxation time, which was measured in each ROI by the method that is also used to estimate the partial volume of cerebrospinal fluid [8], to assign a water concentration. Several groups have independently shown that the T_2_-relaxation time is generally indicative for the water content of the developing brain [41]–[43]. Above (**A**) the T_2_-relaxation times measured in term infants as a function of PC age are shown for parieto/occipital grey matter. Data points were fitted with an exponential function and compared with published data for the water content of the developing human brain. Of note, in independent publications the maximum brain water content is approximately 92% [37], [38], [40]. On the other hand, the highest T_2_-relaxtion times measured with our assay were around 400 ms. From that information a look-up table was constructed as illustrated in graph **B**. Using this methodology we found that the T_2_-relaxation times were slightly lower in the white matter of preterm infants when compared with term infants at equivalent PC age resulting in a slightly lower water content (by ≈1.5%). However, these differences, albeit significant, are very subtle and have no impact on the overall findings and are thus not discussed in more detail in this manuscript. Indeed, in hindsight, a simplified analysis with a constant average water concentration (i.e. 86%) for all spectra for all regions would have minimally altered the time courses of the metabolites versus PC age curves for both term and preterm infants but it would not have altered the findings of this study.
(TIF)

Table S1 **Modeling of Metabolite concentrations versus post-conceptional age in term-born infants.**
(DOCX)
