# Correction: Metabolic Maturation of White Matter Is Altered in Preterm Infants

**DOI:** 10.1371/journal.pone.0091460

**Published:** 2014-02-28

**Authors:** The PLOS ONE Staff

Table S1 is incorrect. Please see a corrected version of Table S1 here:

## Supporting Information

Table S1 Modeling of Metabolite concentrations versus post-conceptional age in term-born infants.
(DOCX)
