# Supplementary figures and images for: Metabolic Maturation of White Matter Is Altered in Preterm Infants
Source: PLoS One. 2014 Jan 22;9(1):e85829. doi: 10.1371/journal.pone.0085829 (PMC3899075; doi:10.1371/journal.pone.0085829)

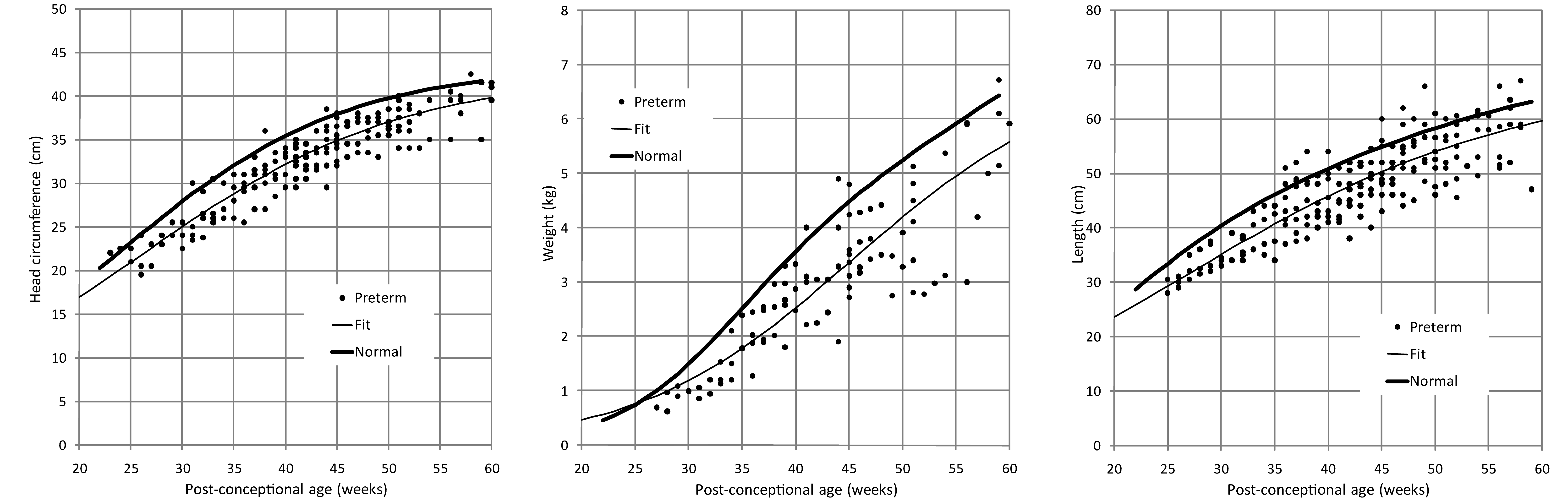

Supplement: Figure S1 — Growth curves for prematurely born subjects. To rule out the possibility that the accelerated WM maturation in the preterm infants was merely a reflection of accelerated somatic growth, we examined serial measurements of head circumference, weight, and body length, recorded from the preterm infants’ medical records. On average, these three measures remained below the expected normal values, but in line with the expected rate of growth (i.e., slope) throughout the study period (i.e., 60 weeks). There was no evidence for a systematic clinical problem with e.g. the head circumference falling further behind over time. This was expected as subjects were selected based on unremarkable clinical follow-up. Note that the number of individual data points exceeds the number of subjects since several measurements (typically weekly) of head circumference (left), weight (center), and body length (right) were taken. The thick solid lines indicate the 50-percintile lines for normal growth (©2003 Fenton TR; licensee BioMed Central Ltd., http://www.biomedcentral.com/1471-2431/3/13). The thin lines represent the best fit of measurements in the preterm cohort. (TIF) [file pone.0085829.s001.tif]

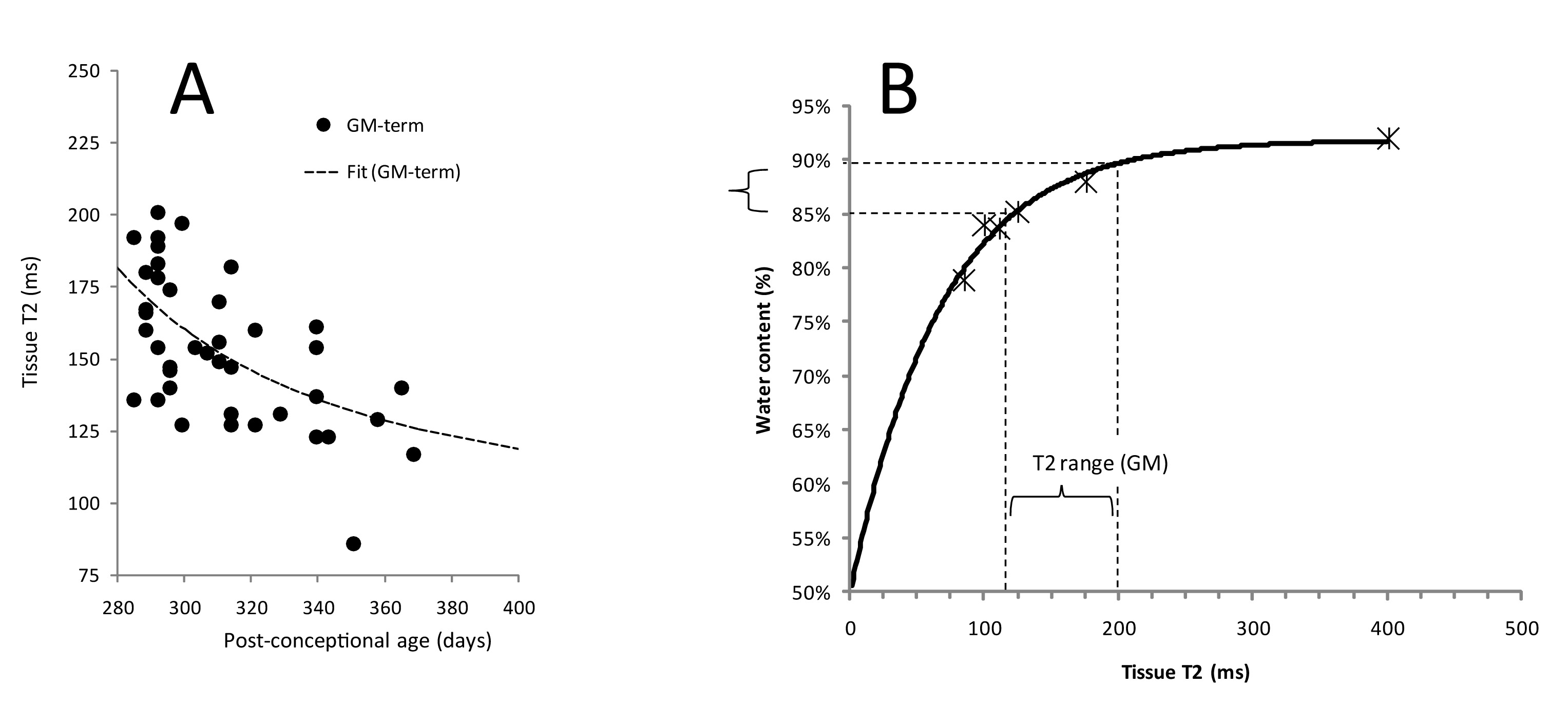

Supplement: Figure S2 — Water concentration. When studying newborns it needs to be considered that brain water concentrations change with age from ≈ 88% at birth to ≈ 85% at six months of age [37]–[40]. However, it is conceivable that brains of term and preterm infants do have different water contents at equivalent PC age. To a priori exclude that differences in water content could possibly be a factor for systematically different metabolite concentrations, we used an approach that does not utilize the age of a subject as the determining factor for the water concentration. Instead, we used the tissue transverse (T2) relaxation time, which was measured in each ROI by the method that is also used to estimate the partial volume of cerebrospinal fluid [8], to assign a water concentration. Several groups have independently shown that the T2-relaxation time is generally indicative for the water content of the developing brain [41]–[43]. Above (A) the T2-relaxation times measured in term infants as a function of PC age are shown for parieto/occipital grey matter. Data points were fitted with an exponential function and compared with published data for the water content of the developing human brain. Of note, in independent publications the maximum brain water content is approximately 92% [37], [38], [40]. On the other hand, the highest T2-relaxtion times measured with our assay were around 400 ms. From that information a look-up table was constructed as illustrated in graph B. Using this methodology we found that the T2-relaxation times were slightly lower in the white matter of preterm infants when compared with term infants at equivalent PC age resulting in a slightly lower water content (by ≈1.5%). However, these differences, albeit significant, are very subtle and have no impact on the overall findings and are thus not discussed in more detail in this manuscript. Indeed, in hindsight, a simplified analysis with a constant average water concentration (i.e. 86%) for all spectra for all regions [file pone.0085829.s002.tif]
