# Supplementary material for: Metabolic Maturation of White Matter Is Altered in Preterm Infants
Source: PLoS One. 2014 Jan 22;9(1):e85829. doi: 10.1371/journal.pone.0085829 (PMC3899075; doi:10.1371/journal.pone.0085829)
Supplement: Table S1 — Modeling of Metabolite concentrations versus post-conceptional age in term-born infants. (DOCX) [file pone.0085829.s003.docx]

|  |  | **Function** | **A_1_** | **A_2_** | **A_3_** |
| --- | --- | --- | --- | --- | --- |
| NAA | Parietal WM | f_1_ | 8.12±0.22 | 11.8±0.80 | 0.81±0.01* |
|  | GM | f_1_ | 12.3±4.5 | 5.62±0.85 | 0.96±0.10 |
|  | Frontal WM | f_1_ | 18.3±21.0 | 4.91±0.75 | 1.06±0.20 |
| Cr | Parietal WM | f_1_ | 6.66±0.30 | 8.85±1.47 | 0.72±0.01 |
|  | GM | f_1_ | 6.35±0.44 | 9.43±2.02 | 0.71±0.01 |
|  | Frontal WM | f_1_ | 6.76±0.98 | 8.48±2.37 | 0.73±0.04 |
| Cho | Parietal WM | f_1_ | 2.17±0.02 | 18.5±5.2 | 0.66±0.03 |
|  | GM | f_2_ | 1.42±0.12 | 0.51±0.15 | - |
|  | Frontal WM | f_2_ | 1.66±0.14 | 0.49±0.16 | - |
| mI | Parietal WM | f_2_ | 18.0±0.6 | -11.3±0.7 | - |
|  | GM | f_2_ | 25.2±0.6 | -17.7±0.7 | - |
|  | Frontal WM | f_2_ | 17.3±0.6 | -10.5±0.6 | - |
| Glu | Parietal WM | f_1_ | 9.10±0.46 | 14.4±1.8 | 0.82±0.10* |
|  | GM | f_1_ | 193±188 | 3.66±0.26 | 1.86±0.14 |
|  | Frontal WM | f_1_ | 891±63 | 2.43±0.25 | 2.91±0.22 |
| Tau | Parietal WM | f_2_ | 3.59±0.34 | -0.96±0.40 | - |
|  | GM | f_2_ | 2.09±0.31 | 1.29±0.37 | - |
|  | Frontal WM | f_2_ | 1.89±0.34 | 1.40±0.40 | - |
