# Supplementary material for: Correction: Metabolic Maturation of White Matter Is Altered in Preterm Infants
Source: PLoS One. 2014 Feb 28;9(2):e91460. doi: 10.1371/journal.pone.0091460 (PMC3945060; doi:10.1371/journal.pone.0091460)
Supplement: Table S1 — Modeling of Metabolite concentrations versus post-conceptional age in term-born infants. (DOCX) [file pone.0091460.s001.docx]

**Table S1: Modeling of Metabolite concentrations versus post-conceptional age in term-born infants.**

Sigmoid (f_1_) and linear functions (f_2_) were used to model metabolite concentrations versus PC age. Fit parameters can only be compared for metabolites that were fitted with the same function. The parameter A_3_ for the sigmoid function denotes the time of the fastest increase of a metabolite. For NAA and Glu there is no overlap of A_3_ for parietal WM with A_3_ for GM or frontal WM. This indicates that the biggest changes of NAA and Glu occurred significantly earlier in parietal WM than in frontal WM or GM. On the other hand no difference was observed for Cr.

|  |  | **Function** | **A_1_** | **A_2_** | **A_3_** |
| --- | --- | --- | --- | --- | --- |
| NAA | Parietal WM | f_1_ | 8.12±0.22 | 11.8±0.80 | 0.81±0.01* |
|  | GM | f_1_ | 12.3±4.5 | 5.62±0.85 | 0.96±0.10 |
|  | Frontal WM | f_1_ | 18.3±21.0 | 4.91±0.75 | 1.06±0.20 |
| Cr | Parietal WM | f_1_ | 6.66±0.30 | 8.85±1.47 | 0.72±0.01 |
|  | GM | f_1_ | 6.35±0.44 | 9.43±2.02 | 0.71±0.01 |
|  | Frontal WM | f_1_ | 6.76±0.98 | 8.48±2.37 | 0.73±0.04 |
| Cho | Parietal WM | f_1_ | 2.17±0.02 | 18.5±5.2 | 0.66±0.03 |
|  | GM | f_2_ | 1.42±0.12 | 0.51±0.15 | - |
|  | Frontal WM | f_2_ | 1.66±0.14 | 0.49±0.16 | - |
| mI | Parietal WM | f_2_ | 18.0±0.6 | -11.3±0.7 | - |
|  | GM | f_2_ | 25.2±0.6 | -17.7±0.7 | - |
|  | Frontal WM | f_2_ | 17.3±0.6 | -10.5±0.6 | - |
| Glu | Parietal WM | f_1_ | 9.10±0.46 | 14.4±1.8 | 0.82±0.10* |
|  | GM | f_1_ | 193±188 | 3.66±0.26 | 1.86±0.14 |
|  | Frontal WM | f_1_ | 891±63 | 2.43±0.25 | 2.91±0.22 |
| Tau | Parietal WM | f_2_ | 3.59±0.34 | -0.96±0.40 | - |
|  | GM | f_2_ | 2.09±0.31 | 1.29±0.37 | - |
|  | Frontal WM | f_2_ | 1.89±0.34 | 1.40±0.40 | - |

$$f_{2}=A_{1}+PCA\cdot A_{2}$$

PCA = post-conceptional age (years).
